# Estrogen Receptor α Mediates Doxorubicin Sensitivity in Breast Cancer Cells by Regulating E-Cadherin

**DOI:** 10.3389/fcell.2021.583572

**Published:** 2021-02-04

**Authors:** Xiaoqing Wan, Jiaxin Hou, Shurong Liu, Yanli Zhang, Wenqing Li, Yanru Zhang, Yi Ding

**Affiliations:** ^1^Laboratory of Molecular Oncology, Weifang Medical University, Weifang, China; ^2^Department of Pathophysiology, Weifang Medical University, Weifang, China; ^3^School of Physical Education & Sports Science, Qufu Normal University, Qufu, China; ^4^Key Laboratory of Applied Pharmacology, Weifang Medical University, Weifang, China

**Keywords:** breast cancer, estrogen receptor α, doxorubicin, chemoresistance, E-cadherin

## Abstract

Anthracyclines resistance is commonly seen in patients with estrogen receptor α (ERα) positive breast cancer. Epithelial-mesenchymal transition (EMT), which is characterized with the loss of epithelial cell polarity, cell adhesion and acquisition of new invasive property, is considered as one of the mechanisms of chemotherapy-induced drug resistance. In order to identify factors that associated with doxorubicin resistance, we performed *in vitro* and *in vivo* experiments using human and mouse breast cancer cell lines with different ERα status. Cell survival experiments revealed that ERα-positive cells (MCF-7 and MCF-7/ADR cell lines), were less sensitive to doxorubicin than ERα-negative (MDA-MB-231, MDA-MB-468) cells, and mouse mammary carcinoma cells (4T-1). The expression of E-cadherin reduced in low-invasive ERα-positive MCF-7 cells after treatment with doxorubicin, indicating epithelial mesenchymal transition. In contrast, the expression of E-cadherin was upregulated in high-invasive ERα-negative cells, showing mesenchymal-epithelial transition (MET). Moreover, it was found that the growth inhibition of 4T-1 cells by doxorubicin was positively correlated with the expression of E-cadherin. In a mouse breast cancer xenograft model, E-cadherin was overexpressed in the primary tumor tissues of the doxorubicin-treated mice. In ERα-positive MCF-7 cells, doxorubicin treatment upregulated the expression of EMT-related transcription factors Snail and Twist, that regulate the expression of E-cadherin. Following overexpression of ERα in ERα-negative cells (MDA-MB-231 and MDA-MB-468), doxorubicin enhanced the upregulation of Snail and Twist, decreased expression of E-cadherin, and decreased the sensitivity of cells to doxorubicin. In contrast, inhibition of ERα activity increased the sensitivity to doxorubicin in ERα-positive MCF-7 cells. These data suggest that the regulation of Snail and/or Twist varies depends on different ERα status. Therefore, doxorubicin combined with anti-estrogen receptor α therapy could improve the treatment efficacy of doxorubicin in ERα-positive breast cancer.

## Introduction

Breast cancer is the most commonly diagnosed cancer among women and the second leading cause of cancer-related death (Bray et al., 2018). Based on tumor ERα status, patients with breast cancer are classified as either estrogen receptor α (ERα) positive or ERα-negative. Nearly 70% of breast cancer patients are ERα-positive, and their tumor growth and development depend on estrogen. There is a significant difference in the sensitivity to chemotherapy between ERα-negative and ERα-positive tumors (Liedtke et al., 2008).

Anthracyclines, such as doxorubicin, used alone or in combination with paclitaxel are the first-line chemotherapeutic regimens for the treatment of breast cancer. Doxorubicin effectively inhibits the synthesis of nucleic acid by intercalating to DNA double helix, leading to tumor cell death. In patients without a pathologically complete response (pCR) after treatment with doxorubicin (i.e., the absence of residual invasive disease in the breast and in the axillary lymph nodes), the residual cancer burden was observed (Symmans et al., 2017). Defining the mechanisms of doxorubicin sensitivity of breast cancer cells may improve the treatment strategy for patients who fail to achieve a pCR.

Epithelial-mesenchymal transition (EMT) is considered to be the initiation and necessary process for tumor metastasis. EMT is a process of cell phenotypic change in which epithelial cells lose their adhesion molecules, such as E-cadherin, and acquire mesenchymal molecules such as N-cadherin and vimentin (Cano et al., 2000). EMT may lead to malignant tumor chemoresistance, particularly seen in pancreatic cancer (Arumugam et al., 2009; Zheng et al., 2015; Elaskalani et al., 2017), lung cancer (Fischer et al., 2015), hepatocellular carcinoma (Dai et al., 2016; Xu et al., 2016), breast cancer (Mallini et al., 2014; Hu et al., 2016; Lambies et al., 2019), and colon cancer (Liu et al., 2015). Several transcription factors such as Snail, Twist, Slug, ZEB, CarB-box-binding factor can regulate this process (Cano et al., 2000; Zhang and Ma, 2012; Lamouille et al., 2014; Jung et al., 2015; Zhang et al., 2015).

Clinically, ERα-negative breast cancer patients are more likely to acquire pCR than ERα-positive patients receiving neoadjuvant chemotherapy with anthracyclines, suggesting that ERα-negative breast tumors are more sensitive to anthracyclines (Liedtke et al., 2008). However, the mechanism of the difference in sensitivity of doxorubicin between ERα-positive and ERα-negative breast cancer patients is not well-understood. In this study, we investigated the role of ERα in doxorubicin sensitivity using five breast cancer cell lines with different ERα status both *in vitro* and *in vivo*.

## Materials and Methods

### Cell Culture

To compare the doxorubicin sensitivity between ERα-positive and ERα-positive breast cancer cells, ERα-positive human breast cancer cell lines (MCF-7 and MCF-7/ADR), ERα-negative human breast cancer cell lines (MDA-MB-231 and MDA-MB-468), and ERα-negative murine breast cancer cell 4T-1 were used in this study. MDA-MB-231, MDA-MB-468 and 4T-1 were obtained from the American Type Culture Collection (ATCC, USA). MCF-7/ADR cells was purchased from Bogu Biology Company (Shanghai, China). MDA-MB-231, MDA-MB-468, and 4T-1 cells were cultured in RPMI-1640 medium. MCF-7 cells were cultured in MEM medium with 0.01 mg/ml insulin. MCF-7/ADR cells were also cultured in RPMI-1640 medium with 0.86 μM doxorubicin. All the media were supplemented with 10% (v/v) heat-inactivated fetal bovine serum (FBS, Gibco, Thermo Fisher Scientific, Waltham, MA), L-glutamine 1% (v/v), penicillin (100 U/ml) and streptomycin (100 U/ml, Solarbio, China). All cells were incubated in a humidified incubator with 5% CO_2_ at 37°C. Doxorubicin (DOX, 25316-40-9, purity 98.0-102.0%) and ICI182780 (129453-61-8, purity ≥98%) were obtained from Sigma-Aldrich.

### MTT Assay

The MTT assay was used to assess the effect of DOX on cell viability. Tumor cells were seeded into 96-well plates with the density of 35~40% for 24 h, starved with 1% FBS medium for 12 h, and then incubated with different concentrations of DOX. After 24 or 48 h, 20 μl 3-(4,5-dimethylthiazol-2-yl)-2,5-diphenyltetrazolium bromide (MTT, M8180, purity≥98%, Solarbio, China) at a concentration of 5 mg/ml was added into each well for 4 h. Medium/MTT mixture was removed and followed by adding 150 μl of DMSO. The light absorbance was measured at 570 nm. The percentage of cell survival rate was calculated using the following formula: survival rate (%) = (OD of treated wells/OD of control well) ×100 (OD, optical density).

### Western Blot

Whole cell protein (25~60 μg) extracted by RIPA lysis buffer from each sample was loaded into 8% or 10% polyacrylamide gel for electrophoresis as previously described (Cheng et al., 2018) followed by membrane transfer carried out at 300 mA for 1.5~2.5 h. Membranes were blocked with 5% non-fat milk in Tris-buffered saline solution (pH 7.4) containing 0.05% Tween-20 and incubated with primary antibodies overnight. The following primary antibodies were used. β-actin (20536-1-AP, Proteintech, China), E-cadherin (24E10, CST, USA), N-cadherin (ab18203, abcam, USA), Estrogen receptor α (ab32063, abcam, USA), Twist (ab49254, abcam, USA), and Snail (L70G2, CST, USA). After incubation with secondary antibodies (A0208, Beyotime, China), the membranes were treated with reagents from the ECL kits (P90719, Millipore, USA) and exposed to X-ray film (Kodak, USA) in darkness.

### Immunofluorescence

Mouse 4T-1 cells were seeded in 24-well plates with the density of 2.5 × 10^4^ for 24 h and then starved with 1% FBS medium for 12 h. Afterward, cells were treated with DOX at different concentrations for 24 h, followed by fixation with 4% paraformaldehyde for 20 min at room temperature. After blocking with goat serum at 37°C for 1 h, cells were incubated with E-cadherin antibody (1:200, 24E10, CST, USA) overnight, and then with TRITC goat anti-rabbit IgG (1:300, ZF-0313, ZSGB-BIO, China) at 37°C for 1 h. After washing with PBS, cell nuclei were stained with DAPI (10 μg/ml, C0065, Solarbio, China) for 10 min at room temperature, and photographed using a Leica fluorescence microscope.

### Plasmid Transfection

Cells were seeded in 6-well plates at the density of 80~90% with the full medium except penicillin and streptomycin for 16 h. Afterward, ESR1 plasmids (2.5 μg, GENE, China) and Lipofectamine 3000 (5 μl, life technologies, USA) were diluted in 125 μl Opti-MEM medium and mixed for 10 min then added to each well. Empty vector was used simultaneously as a negative control. Twelve hours later, the transfection mixture was replaced with fresh medium and protein was extracted after additional 12 h and detected using Western blot to verify the ER expression level in ESR1 plasmids-transfected cells.

### Animal Studies

Six-week-old female BALB/c mice (Shandong University Animal Center, China) housed under specific pathogen free conditions were used for *in vivo* animal experiments. Murine breast cancer 4T-1 cells transfected with GFP (1 × 10^6^ cells) were inoculated into the mammary fat pad of BALB/c mice. After 1 week, 12 mice were randomly divided into two groups. Six mice per group. Animals in the experimental group were given DOX treatment by intraperitoneal injection at a dose of 2.5 mg/kg, once a week. The mice in the other group received 0.9% NaCl as parallel control. After 4 weeks of treatment, the primary breast cancer lesions of both groups were collected and fixed in 10% neutral buffered formaldehyde. All animals used were under an approved protocol of the Institutional Animal Care and Use Committee of Weifang Medical University.

### Immunohistochemistry (IHC)

A 4 μm thick tissue sections were cut from the paraffin-embedded tissues. After dewaxing and rehydration, 3% hydrogen peroxide was used to block endogenous peroxidase. Non-specific binding was blocked with normal goat serum for 1 h at 37°C. Sections were incubated with anti-E-cadherin antibody (1:200, 24E10, CST) overnight at 4°C. The slides were incubated with Solution I (PV9001, Zsbio, China) for 40 min at 37° C and then incubated with Solution II (PV9001, Zsbio, China) for 40 min at 37°C according to the manufacturer's instruction. After washing with PBS, it was developed with DAB (CW0125, CWBIO, China). The slides were counterstained with Mayer's hematoxylin, washed, dehydrated, and the coverslips were mounted with neutral glue.

### Statistical Analysis

Each experiment was repeated at least three times independently. Data statistics are expressed as the mean ± SD of the specified number of individual experiments. Statistical analysis was performed with GraphPad Prism software (version 5.01, San Diego, CA). ANOVA (parametric) test was used for multiple comparisons and Student's *t*-test was used for two-group comparisons. *P* < 0.05 was considered as statistically significant.

## Results

### The Cytotoxic Effect of DOX Is Associated With Expression of ERα in Breast Cancer Cells

**Figure 1:**
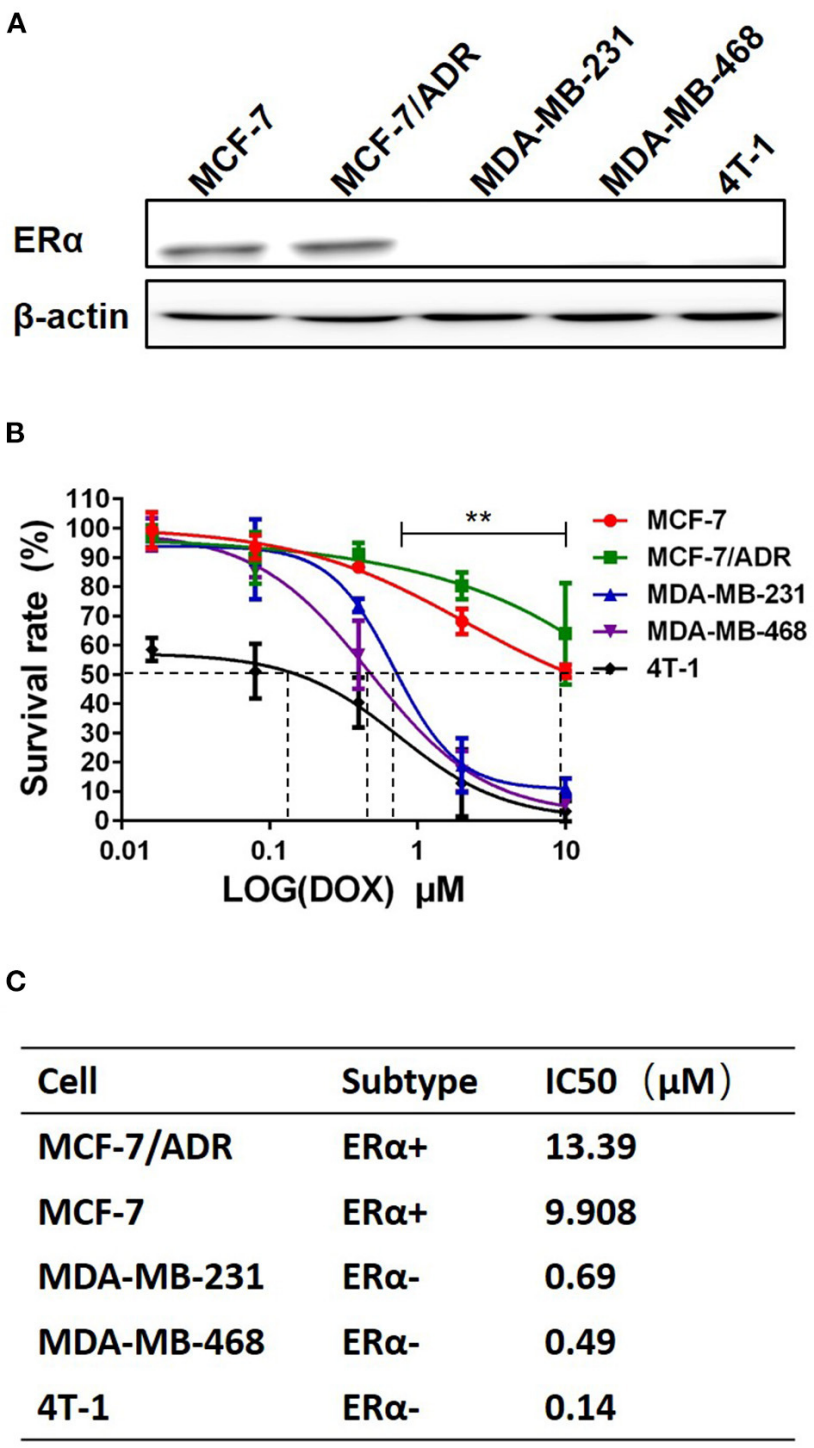
ERα-positive breast cancer cells are less sensitive to DOX than ERα-negative cells. **(A)** ER**α** of breast cancer cell lines of five different molecular subtypes was detected by Western blot. **(B)** Five breast cancer cell lines were incubated with DOX (0, 0.016, 0.08, 0.4, 2 and 10 μM) for 48 h, and DOX cytotoxicity was measured by MTT assay. **(C)** The IC50 of DOX for each cell line was calculated by Graphpad prism 5. Difference of IC50 of these 5 cells were analyzed by student's *t*-test. **P < 0.01.

To study the cytotoxic effects of DOX on breast cancer cells, five breast cancer cell lines, including MCF-7, MCF-7/ADR, MDA-MB-231, MDA-MB-468 and 4T-1, were used. The ERα expression in these five breast cancer cell lines was examined by western blot to confirm the cell subtype. The expression of ERα was seen in MCF-7 and MCF-7/ADR cells, but not in MDA-MB-231, MDA-MB-468 and 4T-1 cells (Figure 1A). These cells were cultured and treated with DOX at different concentration (0–10 μM) for 48 h. It was found that DOX inhibited cell survival in a dose-dependent manner. MCF-7 and MCF-7/ADR cells were less sensitive to DOX than MDA-MB-231, MDA-MB-468 and 4T-1 cells (Figure 1B). The IC50 of DOX in MDA-MB-231, MDA-MB-468 and 4T-1 cells were 0.69, 0.49, and 0.14 μM, respectively, which were lower than that in MCF-7 and MCF-7/ADR cells (9.908 and 13.39 μM, respectively, Figure 1C). The difference of sensitivity to DOX between ERα-negative and ERα-positive breast cancer cells is statistically significant. This result suggests that different sensitivity of cells to DOX is associated with the presence or absence of ERα. ERα-negative cells are more sensitive to DOX than ERα-positive cells.

### DOX Regulates EMT in Human Breast Cancer Cells

To address whether EMT is involved in sensitivity to DOX treatment in breast cancer cells, cells with different phenotypes and ERα expression were incubated with DOX and the related epithelial and mesenchymal phenotypic biomarkers were characterized using western blots.

**Figure 2:**
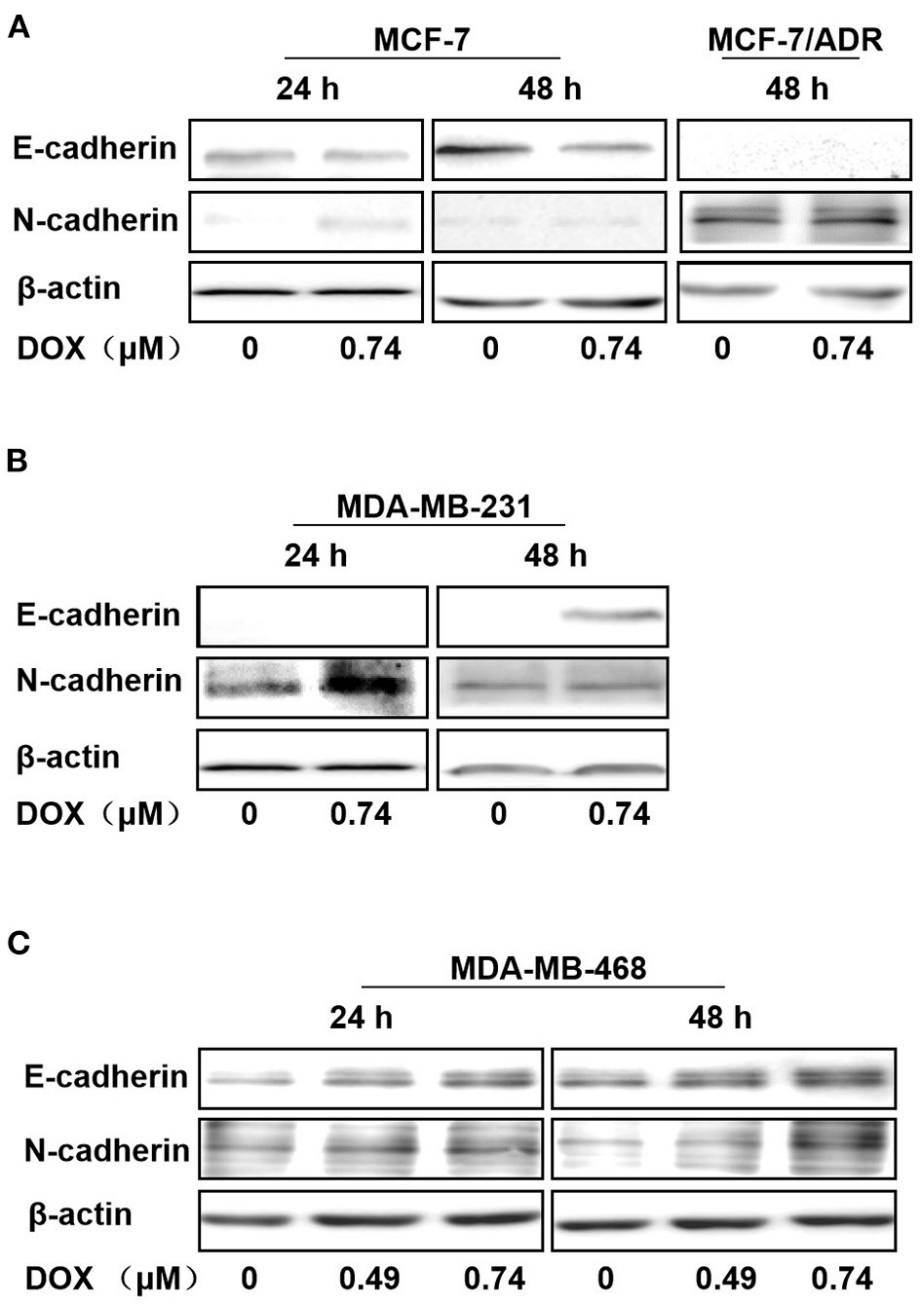
Regulation of E-cadherin, N-cadherin by DOX in ERα-positive and ERα-negative human breast cancer cells. **(A)** MCF-7 cells were treated with DOX (0, 0.74 μM) for 24 and 48 h, and MCF-7/ADR cells were treated for 48 h. **(B)** MDA-MB-231 cells were treated with DOX (0, 0.74 μM) for 24 and 48 h. **(C)** MDA-MB-468 cells were treated with DOX (0, 0.49, 0.74 μM) for 24 and 48 h. Cells were then harvested and expression of E-cadherin, N-cadherin and β-actin was detected by Western blot.

In ERα-positive MCF-7 cells, E-cadherin was downregulated with 0.74 μM DOX (Gu et al., 2016) incubation for 24 and 48 h, and N-cadherin was upregulated by DOX treatment for 24 and 48 h (Figure 2A). In contrast, in ERα-positive MCF-7/ADR, E-cadherin expression was too low to be detected with or without DOX incubation (Figure 2A). In ERα-negative MDA-MB-231 cells, E-cadherin couldn't be detected at 24 h with or without DOX, but was upregulated with DOX treatment for 48 h (Figure 2B). Interestingly, N-cadherin was upregulated at 24 h with DOX treatment, while no obvious difference at 48 h (Figure 2B). Similarly, in ERα-negative MDA-MB-468 cells, both E-cadherin and N-cadherin expression were upregulated in a dose-dependent manner with DOX incubation for 24 and 48 h (Figure 2C).

Therefore, N-cadherin expression was increased with DOX treatment in ERα-positive MCF-7, ERα-negative MDA-MB-231 and MDA-MB-468 cells, while E-cadherin was increased in MDA-MB-231 and MDA-MB-468 cells and decreased in MCF-7 cells. E-cadherin was not detected in MCF-7/ADR cells even after 48 h incubation with DOX. These data suggest that DOX promotes EMT primarily in ERα positive cells and this may contribute to its chemo-resistance.

### DOX Upregulates E-Cadherin Expression in Mouse ERα-Negative Breast Cancer Cells *in vitro* and *in vivo*

**Figure 3:**
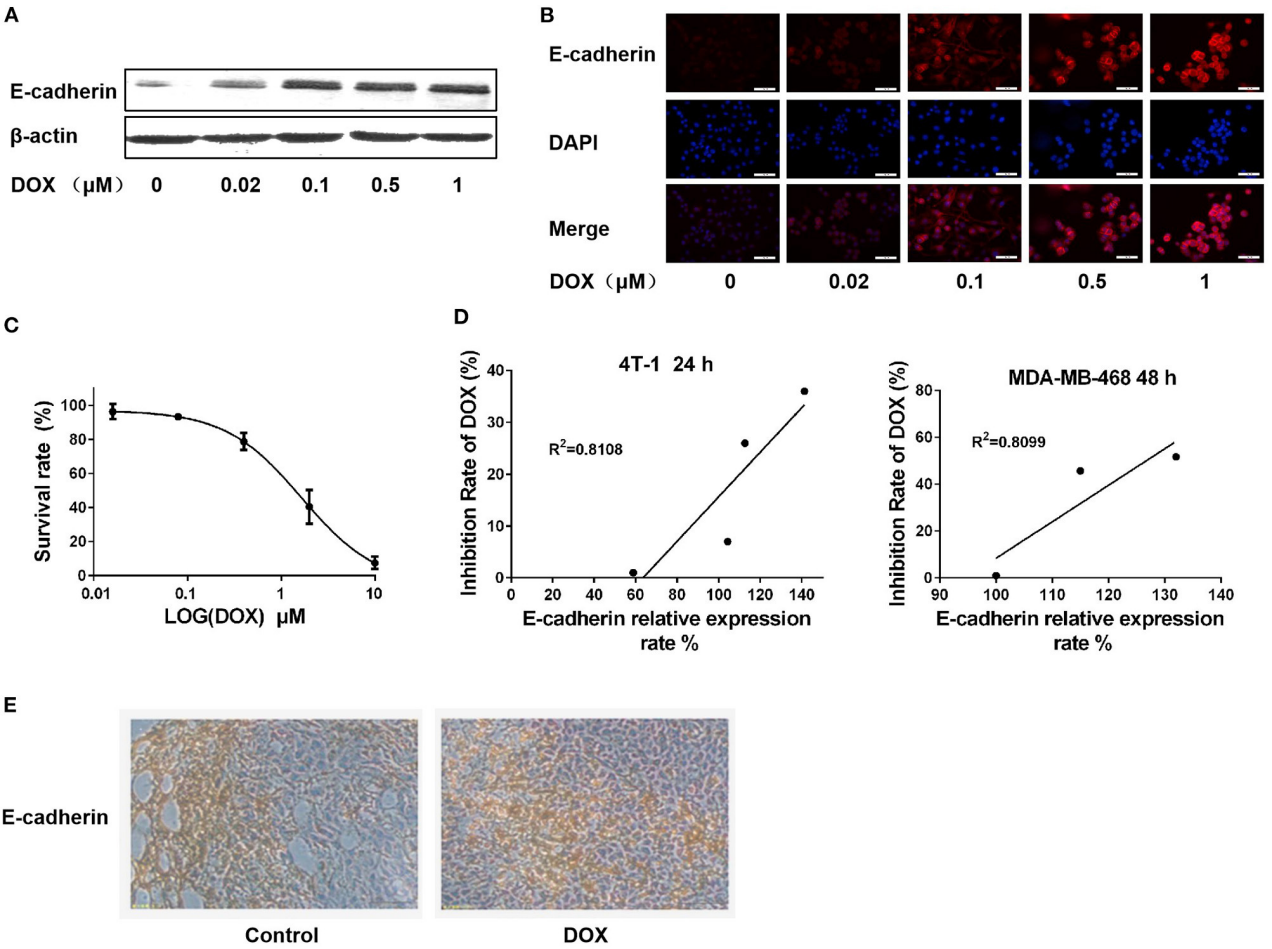
DOX upregulates E-cadherin expression in 4T-1 cells *in vitro* and *in vivo*, and expression of E-cadherin is positively correlated with cell sensitivity to DOX. **(A)** 4T-1 cells were treated with DOX (0, 0.02, 0.1, 0.5, and 1 μM) for 24 h. The cells were harvested and expression of E-cadherin and β-actin were detected by Western blot. **(B)** 4T-1 cells were treated with DOX (0, 0.02, 0.1, 0.5, and 1 μM) for 24 h. The expression of cellular E-cadherin detected by immunofluorescence assay. **(C)** 4T-1 cells were incubated with DOX (0, 0.016, 0.08, 0.4, 2, and 10 μM) for 24 h, and DOX cytotoxicity was measured by MTT assay. **(D)** Correlation analysis on the relationship between cell inhibition rate and E-cadherin expression in 4T-1 and MDA-MB-468 cells. **(E)** BALB/c mouse was inoculated with 4T-1 cells in the subcutaneous fat pad of mammary gland and intraperitoneally administrated with DOX (0 and 2.5 mg/Kg) for 4 weeks. E-cadherin expression in primary breast cancer tissues was detected by IHC.

The mouse ERα-negative breast cancer 4T-1 cells were cultured and treated with different concentrations of DOX for 24 h. The expression of E-cadherin was determined by western blot and immunofluorescence assay. Both western blot and immunofluorescence assay results revealed that E-cadherin was upregulated in a dose-dependent manner (Figures 3A,B), which is in consistent with results in the human ERα-negative breast cancer MDA-MB-231 and MDA-MB-468 cells.

The cytotoxic effect of DOX on 4T-1 cells was assessed by MTT assay. After 24 h of DOX incubation, the survival rate of 4T-1 cells was reduced in a dose-dependent manner (Figure 3C). The correlation between E-cadherin expression and DOX proliferation inhibition was analyzed in both mouse and human ERα-negative breast cancer cells (4T-1 and MDA-MB-468) to clarify whether the cytotoxicity of DOX on breast cancer cells is associated with expression of E-cadherin. The results showed that there was a significant positive correlation between the cytotoxicity of DOX and the expression of E-cadherin (Figure 3D) suggesting that the chemosensitivity of breast cancer cells to DOX is related to E-cadherin expression.

BALB/c mice were used for 4T-1 breast cancer model *in vivo* studies. After 4 weeks of intraperitoneal injection of DOX (2.5 mg/kg) or the same amount of physiological saline as a control, primary cancer tissues of each group was isolated, and E-cadherin expression was detected by IHC. Consistent with our *in vitro* results, the expression of E-cadherin in the DOX treatment group was higher than in the control group (Figure 3E).

### DOX Upregulates the Expression of Snail and Twist in Breast Cancer Cells

**Figure 4:**
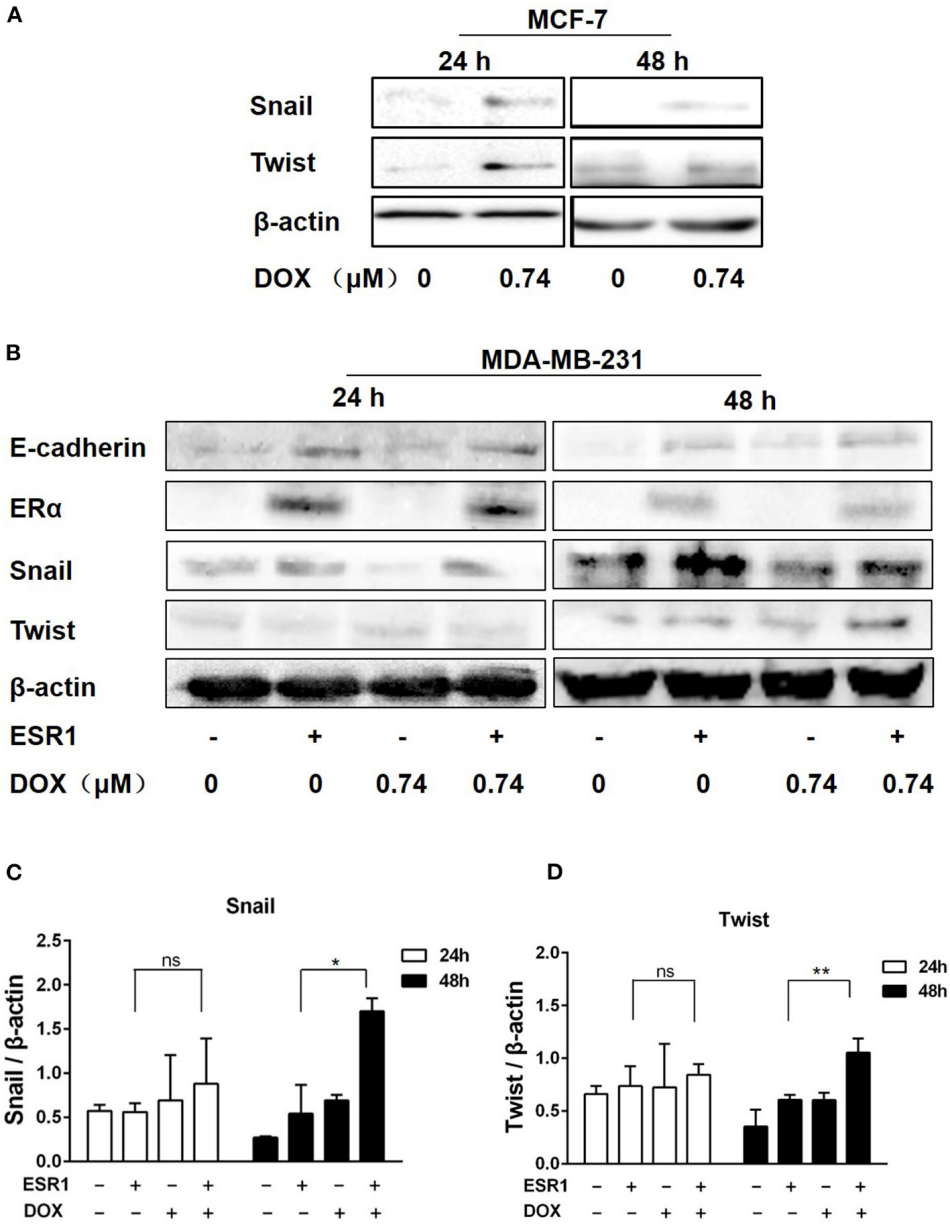
DOX induced the expression of Twist and Snail in breast cancer cells. **(A)** MCF-7 cells were treated with DOX (0 and 0.74 μM) for 24 and 48 h. Cells were then harvested and the expression of Snail, Twist and β-actin was determined by Western blot. **(B)** MDA-MB-231 cells were transfected with the ESR1 plasmid or the negative control plasmid, followed by treatment with DOX (0 and 0.74 μM) for 24 and 48 h. Cells were then harvested and the expression of E-cadherin, ER**α**, Snail, Twist and β-actin was detected by Western blot. **(C,D)** The relative expression levels of Snail and Twist proteins were analyzed based on gray intensity. *P < 0.05 vs. ER**α** overexpressed cells without DOX. **P < 0.01.

Snail and Twist are key molecules regulating EMT process. The expression of both Snail and Twist were upregulated in ERα-positive MCF-7 cells treated with DOX (0.74 μM) for 24 and 48 h (Figure 4A). In order to further delineate the findings in MCF-7 cells and MDA-MB-231 cells, these cells were transfected with ERα (ESR1) plasmid or empty vector as a negative control. After treatment with or without DOX (0.74 μM) for 24 or 48 h, the expression of the ERα was significantly increased in ERα (ESR1) plasmid transfected cells (Figure 4B).

Snail was upregulated by DOX treatment either in ERα overexpressing cells or controls (Figures 4B,C). After 48 h of DOX treatment, Snail increased in ERα overexpressing cells, and ERα overexpression enhanced DOX-induced Snail upregulation (Figures 4B,C). Similarly, Twist upregulation of ERα at 48 h and a synergistic effect of DOX and ERα on Twist expression was found (Figures 4B,D).

E-cadherin was upregulated in the presence of ERα and this effect was attenuated by DOX treatment for 24 and 48 h (Figure 4B). These data indicate that, DOX may promote EMT by upregulating Snail and Twist via an ERα related mechanism.

### The Cytotoxic Effect of DOX Is Related to ERα Expression in Breast Cancer Cells

**Figure 5:**
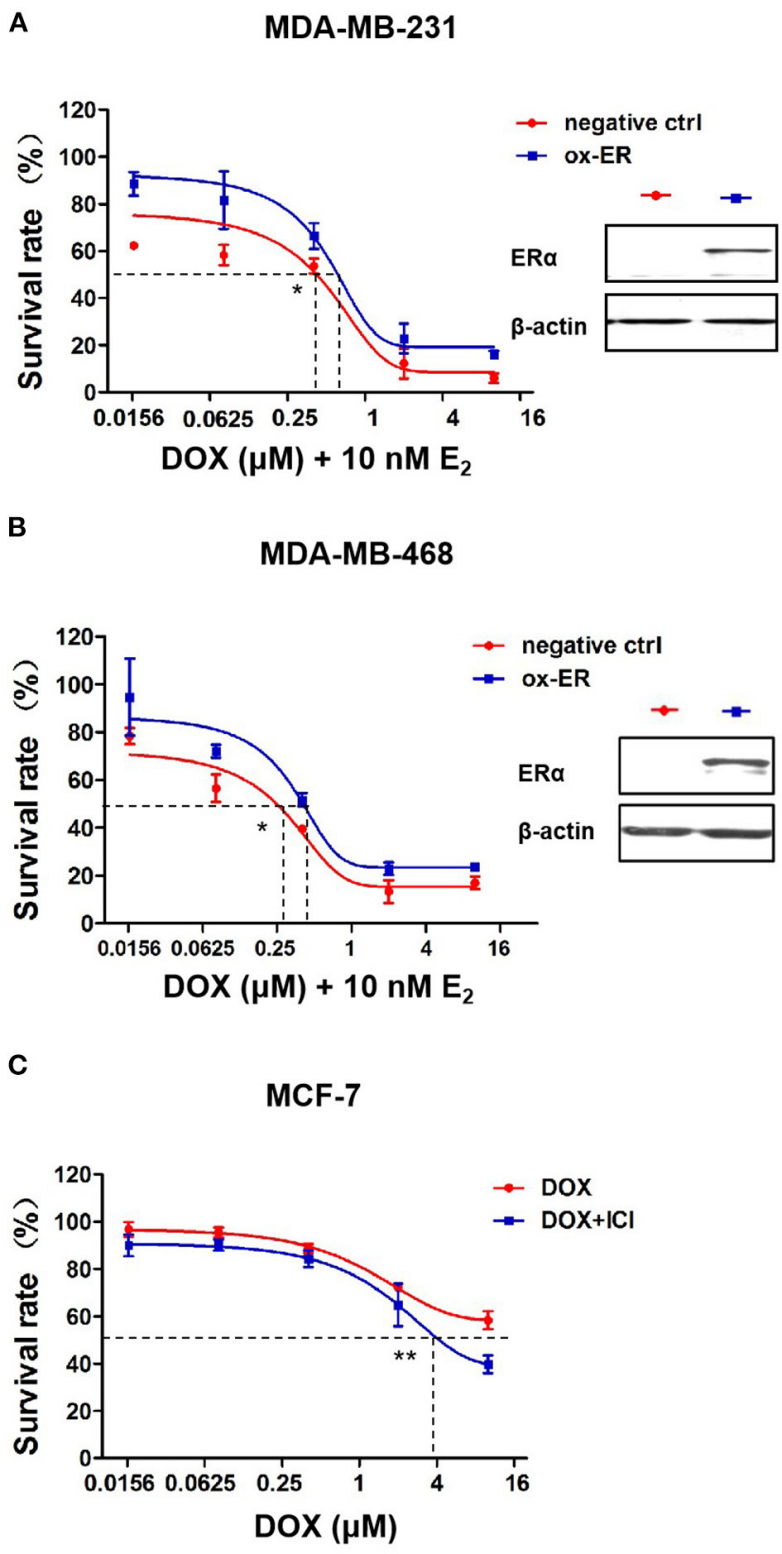
ERα affects the sensitivity of breast cancer cells to DOX. **(A)** MDA-MB-231 cells and **(B)** MDA-MB-468 cells transfected with ESR1 plasmid or negative control plasmid, followed by DOX treatment at 0, 0.016, 0.08, 0.4, 2, and 10 μM for 48 h. DOX cytotoxicity was measured by MTT assay. **(C)** MCF-7 cells were treated with DOX (0, 0.016, 0.08, 0.4, 2, and 10 μM) alone or in combination with ICI (1 μM, a specific inhibitor of ER**α**) for 48 h. Drug cytotoxicity was detected by MTT assay. Difference of IC50 of each cell line were analyzed by student's *t*-test. *P < 0.05, **P < 0.01.

To identify the role of ERα in chemotherapeutic resistance to DOX, MDA-MB-231 and MDA-MB-468 cells were transfected with either an ERα plasmid or an empty vector as a negative control. After transfection, MDA-MB-231 and MDA-MB-468 cells were incubated with DOX plus 10 nM estradiol, an activator of ERα signaling. The cell survival rate is higher in the ERα overexpression group than that in the MDA-MB-231 (Figure 5A) and MDA-MB-468 cells (Figure 5B) after DOX treatment. Furthermore, for MDA-MB-231 cells, the IC50 of DOX in cells of the negative control group was 0.39 vs. 0.61 μM in ERα overexpression group. And for MDA-MB-468 cells, the IC50 of DOX in cells of the negative control group was 0.26 vs. 0.41 μM in ERα overexpression group. The difference of IC50 in each cell line is statistically significant.

By incubating MCF-7 ERα positive cells with 1 μM ICI182780, a specific inhibitor of ERα, ERα signaling was inhibited and MCF-7 cells became more sensitive to DOX than cells without ICI182780 treatment (Figure 5C). These data suggest that ERα plays an important role in mediating the chemo sensitivity of breast cancer cells to DOX treatment.

## Discussion

Patients with ERα-negative breast cancer are more sensitive to anthracyclines-based neoadjuvant chemotherapy than ERα-positive patients (Liedtke et al., 2008), but the mechanism behind this response remains uncleared. In this study, we have observed an association of DOX cytotoxicity with expression of EMT biomarkers in multiple breast cancer cells with different ER status.

### DOX Resistance Is Positively Correlated With ERα and E-Cadherin Expression *in vitro* and *in vivo*

ERα-negative breast cancer cells (MDA-MB-231, MDA-MB-468 and 4T-1 cells) were more susceptible to the cytotoxic effect of DOX than ERα-positive (MCF-7 and MCF-7/ADR) cells. It was reported that although ERα-negative breast cancer has more mesenchymal characteristics and poorer prognosis than ERα-positive tumors (Tomaskovic-Crook et al., 2009), ERα-negative tumor subtypes are more likely to be benefited from DOX treatment than the other breast cancer subtypes.

The chemo-resistance of breast cancer cells to DOX depends in part on the expression of E-cadherin. Our study demonstrated that the expression of E-cadherin is downregulated by DOX treatment in MCF-7 cells. DOX-resistant MCF-7/ADR cells have very low level of E-cadherin. In contrast, E-cadherin was upregulated by DOX treatment in the DOX sensitive MDA-MB-231 and MDA-MB-468 human breast cancer cells. Similar upregulation of E-cadherin by DOX treatment was seen in the mouse 4T-1 cells.

Furthermore, we found a positive correlation between DOX cytotoxicity and expression of E-cadherin in 4T-1 cells, which is in consistent with the observations by Zhou et al. (2014) and Wang et al. (2017). In our BALB/c mouse tumor model, expression of E-cadherin was increased in primary tumor compared to controls. *In vitro* studies by other investigators have also shown that MCF-7 cells are more resistant to DOX and have mesenchymal characteristics after E-cadherin knockout (Zhou et al., 2014). These data suggest that E-cadherin may be a useful biomarker for determining the susceptibility of breast cancer to DOX therapy.

### The Effect of DOX on E-Cadherin Expression Is Mediated by EMT-Related Transcription Factors in Breast Cancer Cells

EMT can increase migration and invasion potential of tumor cells by increasing expression of mesenchymal biomarkers, N-cadherin, vimentin and fibronectin and decreasing epithelial cell phenotype biomarkers like E-cadherin (Cano et al., 2000). Overexpression of E-cadherin in tumor cells prevents transcription of mesenchymal genes (Ohkubo and Ozawa, 2004; Solanas et al., 2008). Recent studies have shown that tumor cells undergoing EMT exhibit enhanced multidrug resistance (MDR) (Fischer et al., 2015; Zheng et al., 2015; Xu et al., 2016; El Amrani et al., 2019). In the current study, DOX reduced E-cadherin expression in MCF-7 cells. MCF-7 cells is more resistant to DOX than MDA-MB-231 cells. Notably, in MDA-MB-231 cells, we found that DOX increased the expression of E-cadherin suggesting that mesenchymal to epithelial transitions may contribute to chemical resistance to DOX.

Previous studies have shown that EMT-induced transcription factors, such as Twist, Snail and Zeb1, as well as some signaling pathways. Transforming growth factor beta, Wnt and Notch, can induce EMT and result in chemoresistance (Moreno-Bueno et al., 2008). E-cadherin is a key protein in the formation of adhesion junctions, and it is essential to maintain the epithelial phenotype by binding to neighboring cells (van Roy and Berx, 2008). In the current study, both Snail and Twist were upregulated by DOX treatment in MCF-7 cells. In MDA-MB-231 cells, Snail and Twist also increased after 48 h of DOX treatment.

The function of E-cadherin could be disrupted in tumor invasion and metastasis (Cano et al., 2000; Arumugam et al., 2009). There are several mechanisms involved in downregulation of E-cadherin during tumor progression such as transcription downregulation, mutation, and methylation (van Roy and Berx, 2008; Cui et al., 2018). EMT-induced transcription factors including Snail, Zeb1 and Zeb2, that can inhibit E-cadherin transcription (Moreno-Bueno et al., 2008). For example, the transcription factors Snail, E12/E47, Zeb1 and SIP1 bind to E-box elements at the proximal promoter site of E-cadherin leading to transcriptional inactivation of E-cadherin. Snail binds to partly overlapping promoter sequences and downregulates the expression of E-cadherin in breast cancer cells (Cano et al., 2000). Twist is possibly involved in EMT by repressing E-cadherin and promoting expression of N-cadherin.

Our data showed that DOX upregulated the expression of Snail and Twist to stimulate the transcriptional expression of E-cadherin and trigger the process of MET. Although DOX induced Snail and Twist expression consistently in MCF-7 cells and MDA-MB-231 cells, these cells showed different responses to DOX treatment in E-cadherin expression. We hypothesized that this might be due to the different expression of ERα and further investigated the role of ERα in DOX-induced Snail and Twist expression.

### The Regulation of E-Cadherin Expression by Snail and Twist Is Associated With ERα in Breast Cancer Cells

Our studies demonstrate that DOX increases Snail and Twist expression, and this effect is significantly enhanced by ERα. We hypothesized that DOX treatment activates Snails and Twist signaling pathways and E-cadherin gene transcription is determined by ERα status in breast cancer cells. In the absence of ERα, Snail triggers E-cadherin gene transcription. However, in ERα-positive cells, ERα may be a cofactor of Snail, Twist or both to inhibit E-cadherin gene transcription, resulting in E-cadherin reduction and EMT. More research is warranted to support this result. It has been reported that ERα signaling is regulated by EMT-associated transcription factors (Ye et al., 2008, 2010; Al Saleh et al., 2011; Vesuna et al., 2012; Bouris et al., 2015). Vesuna et al. (2012) et al. found that Twist binds to the E-box in the ERα promoter, thereby inhibiting the expression of ERα. A Twist overexpressing MCF-7 cells mouse breast cancer model showed resistance to endocrine therapy with tamoxifen (ATM). And suppression of Twist can partially restore the function of ERα. Therefore, in ERα-positive breast cancer cells, DOX treatment can upregulate Snail and Twist leading to EMT, and suppress ERα expression can attenuate the sensitivity of cells to ERα therapy.

In clinical settings, patients with ERα-positive breast cancer and high expression of Twist and Snail usually indicate a high risk of tumor recurrence (van Nes et al., 2012). In addition, previous studies suggested that Twist can induce Snail, and the expression of Snail is an important biomarker indicating Twist activation (Ip et al., 1992; Smit et al., 2009). Therefore, the increase of Snail may be partially affected by increasing Twist. Other transcription factors such as Zeb1/2 and Slug can downregulate E-cadherin by direct binding to its promoter (Ye et al., 2010; Kurahara et al., 2012). In the case of Snail and Twist upregulation, the upregulation of E-cadherin in MDA-MB-231 cells in response to DOX treatment may due to the effect of other transcription factors. The specific mechanism needs to be further explored.

**Figure 6:**
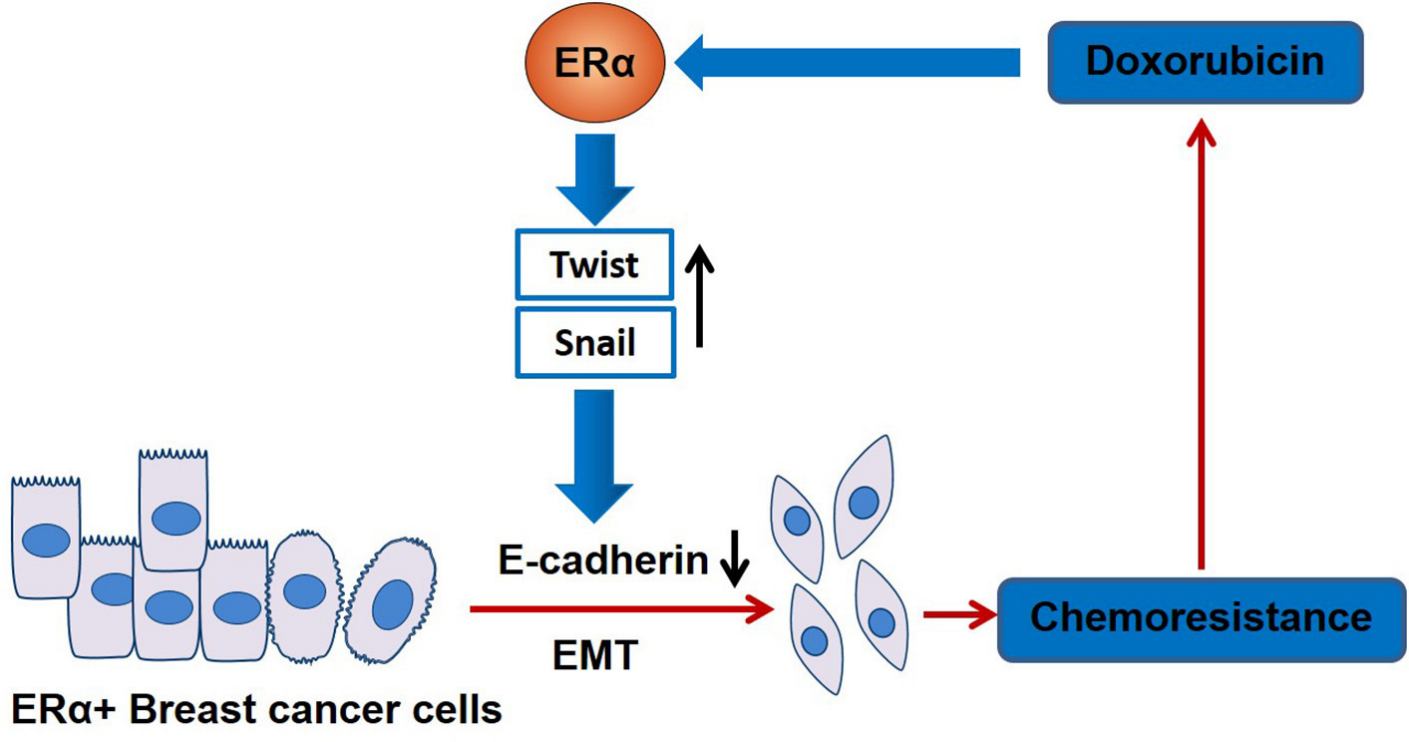
A schematic mechanism of DOX chemoresistance in ERα-positive breast cancer cells. In ER**α**-positive breast cancer cells, DOX treatment switches on ER**α**-mediated EMT pathway. Through ER**α**, DOX increases EMT key transcription factors, Snail and Twist, resulting in the decrease of E-cadherin and promoting EMT in ER**α**-positive breast cancer cells. Cells undergoing EMT exhibit DOX-resistance.

In summary, our work suggests that DOX treatment promotes EMT leading to chemo-resistance in ERα-positive breast cancer through the ERα signaling pathway (Figure 6). This might be the reason why patients with ERα-negative breast cancer are easier in obtaining pCR than those with ERα-positive cancer after anthracycline-based chemotherapy. Therefore, therapeutic strategy using alternative chemotherapeutic drugs or combining an ERα-target inhibitor can benefit patients with ERα-positive breast cancer by avoiding drug resistance.

## Novelty and Impact

Anthracyclines, the first-line chemotherapeutic drugs for the treatment of breast cancer, are prone to drug resistance in patients with positive estrogen receptor α (ERα). However, the mechanism of drug resistance is largely unknown. We performed both *in vitro* and *in vivo* studies using breast cancer cells with different ERα status by overexpression and inhibition of ERα. It was found that doxorubicin treatment upregulates the expression of transcription factors that related to epithelial-mesenchymal transition (EMT), leading to different drug sensitivity depending on the ERα status of the cells. Our results suggest that ERα could be a switch that regulates EMT or MET in the action of doxorubicin.

## Data Availability Statement

The raw data supporting the conclusions of this article will be made available by the authors, without undue reservation.

## Ethics Statement

The animal study was reviewed and approved by Ethics Committee of Weifang Medical University.

## Author Contributions

YD designed and developed the study. XW, JH, SL, YanlZ, WL, and YanrZ performed experiments. XW and YD analyzed the data and wrote the manuscript. All authors reviewed and approved the final manuscript.

## Conflict of Interest

The authors declare that the research was conducted in the absence of any commercial or financial relationships that could be construed as a potential conflict of interest.

## Abbreviations

ERα: Estrogen receptor α
DOX: Doxorubicin
EMT: Epithelial-mesenchymal transition
BC: Breast cancer
E2: estradiol
ICI: ICI182780
ox-ER: over-expression ERα
pCR: pathologically complete response.
